# Pancreas transplantation with grafts obtained from donation after cardiac death or donation after brain death results in comparable outcomes

**DOI:** 10.3389/frtra.2023.1176398

**Published:** 2023-08-09

**Authors:** Michael S. Bleszynski, Catherine Parmentier, Alejandro Torres-Hernandez, Samrat Ray, Anila Yousuf, Andrea Norgate, Jeffrey Schiff, Chaya Shwaartz, Gonzalo Sapisochin, Ian McGilvray, Markus Selzner, Trevor W. Reichman

**Affiliations:** Ajmera Transplant Centre, Toronto General Hospital, Toronto, ON, Canada

**Keywords:** pancreas transplantation, donation after brain death (DBD), donation after cardiac death (DCD), simultaneous kidney and pancreas transplant (SPK), pancreas after kidney transplant (PAK), pancreas transplant alone (PTA), delayed graft function (DGF)

## Abstract

**Introduction:**

Pancreas organ shortages and long recipient waitlist times are critical components that limit recipients from receiving a pancreas transplant. Over the last decade, our center has been using donation after cardiac death (DCD) donors as an adjunct to donation after brain death (DBD) donors to expand the organ pool. The aim of this study was to compare recipient and graft survival between DCD and DBD recipients.

**Methods:**

A retrospective single center propensity matched analysis (2011–2020) of 32 DCD vs 96 DBD pancreas transplants was performed.

**Results:**

8-year recipient survival was similar between DCD and DBD groups (87.4% vs 92.7%, *p*=0.35) as was simultaneous kidney and pancreas transplant (SPK) 8-year kidney (88.9 vs 96.9%, *p*=0.219) and pancreas graft survival (77.4% vs 86.7%, *p*=0.344). There was no difference in vascular thrombosis rate between DCD and DBD pancreas grafts (3.1% vs 7.3%, *p*=0.73). DCD kidneys had a higher rate of DGF vs DBD kidneys (28.1% vs 6.3%, p=0.004), without any significant difference in long term kidney failure (12.5% vs 8.3%, *p*=0.5).

**Discussion:**

Recipients of DCD grafts demonstrate equivalent long-term patient and graft survival compared to DBD recipients for pancreas transplantation. Increased utilization of well selected DCD donors is a safe strategy to increase the donor pool.

## Introduction

Simultaneous pancreas and kidney (SPK) transplantation remains the gold standard treatment for insulin dependent diabetic patients with end-stage renal disease (ESRD) (1–3). SPK provides long-term insulin independence, normoglycemia, stabilization of diabetic complications, reduced incidence of cardiac events and improved patient survival (4). SPK transplantation provides a significant survival advantage over patients who remain on the waitlist or receive a deceased donor kidney transplant alone (KTA) (5). Recipients of pancreas after kidney (PAK) transplantation (following either a living donor or deceased donor kidney transplant) also benefit from improved kidney survival and quality of life compared to KTA (3, 6). Recent evidence has also demonstrated that type 1 diabetes mellitus (T1DM) patients with ESRD who receive a living donor kidney followed by a pancreas transplant may have similar outcomes compared to SPK recipients (2).

Over the last decade, the rates of pancreas transplantation have continued to decline, despite continued improvement in patient and graft outcomes (7, 8). Waitlist times for potential SPK recipients continue to grow due to the imbalance of available and perceived suitability of pancreas donor organs. In the USA, approximately 23%–30% of pancreas donor grafts are discarded depending on the year (8, 9). In 2020, pancreas discard rates among donors aged 40–54 increased to 56% from 27% in 2018 (8). Furthermore, donation after cardiac death (DCD) pancreas utilization remains quite low, accounting for less than 5% of pancreas transplants (10). The declining rate of pancreas transplantation is thought to be, in part, due to the lack of optimal donor organs (11). Surgeons remain wary of the potential risk of using DCD SPK organs due to concerns of pancreatic cellular injury during donor warm ischemia time (WIT), graft injury during procurement, risk of graft thrombosis, and/or pancreatitis within the recipient (12).

Donation after brain death (DBD) remains the predominant source of pancreas organs. Extended donor criteria including BMI > 30 kg/m^2^, age >50, and use of DCD donors have been introduced with the goal of expanding the pancreas donor pool (9). An increased pool of DCD organs could help offset the organ shortage in North America, as seen in parts of Europe (9). The UK has an extensive experience with using DCD pancreata – 30% of pancreas transplants are performed using a DCD pancreas graft (13).

Despite the increased utilization of DCD pancreas allografts in the UK, there remains a need to capture long-term outcomes of patient and graft survival in DCD pancreas transplantation (14, 15). The primary objective of our study was to evaluate long-term graft and recipient survival between DBD and DCD donors at a large volume single Canadian center. Secondary objectives were to assess recipient morbidity and peri-operative complications.

## Materials and methods

Between January 2011 and December 2020, 600 pancreas transplants (SPK, PAK, and PTA) were performed at our center, with 32 DCD pancreas donors being used for transplantation. To compare recipient outcomes between DCD and DBD donors we performed a retrospective propensity matched analysis with a 1:3 ratio of DCD to DBD donors. Propensity matching was based on the type of transplant performed (SPK vs. PAK), date of transplant and recipient indication (T1DM or type 2 diabetes mellitus (T2DM)). Data was collected via a prospectively maintained database. Missing and/or additional variables were collected retrospectively. No DCD pancreata were procured for PTA transplants, therefore DBD donors for PTA transplant were not included for propensity matching. The University Health Network (UHN), Board of Ethics approved this study.

### Selection criteria

Recipient inclusion criteria included those undergoing SPK, PAK, recipients with prior pancreas transplant and patients with T1DM or T2DMN. One DBD SPK recipient was a re-transplant case, matched to a DCD SPK recipient. DCD grafts were only retrieved from controlled Maastricht type 3 DCD donors. Exclusion criteria were pediatric recipients defined as those under 18 years old and recipients of another organ other than SPK or PAK.

### Variables

Donor demographics that were collected include: age, gender, BMI, and cause of death. Recipient demographics that were collected include age, gender, BMI, transplant indication (T1DM or T2DM), HBA1c levels, time on waitlist, and time on dialysis. Operative variables collected were type of pancreas transplant (SPK, PAK); pancreas/kidney cold ischemia time (CIT) and warm ischemia time (WIT); operation duration; and need for re-laparotomy (including graft pancreatectomy). Post-operative outcome data that were collected included pancreas graft failure; post-operative complications (infections, anastomotic bowel leak, pancreatitis, acute/chronic rejection, vascular thrombosis); graft/recipient survival; hospital length of stay; ICU length of stay; duration of long-term recipient follow-up; delayed graft function (DGF) of the kidney; and kidney graft survival.

### Definitions

Pancreas graft failure: recipients who underwent graft pancreatectomy, return to exogenous insulin, re-listing for pancreas transplant, or recipient death (due to any cause).

Post-transplant T2DM: requirement of oral hypoglycemic agents post pancreas transplant, without the need for exogenous insulin.

Pancreas rejection: defined as per Banff classification (16).

DGF of the kidney: dialysis required within the first post-operative week.

DCD donor WIT: time of donor withdrawal from life support (WLS) to time of cold perfusion.

Pancreas/kidney CIT: time from cold perfusion to time of warm reperfusion of the donor graft within the recipient.

Pancreas/kidney WIT: the time of the donor graft removal from the cold to time of warm perfusion (within the recipient).

Functional WIT (f-WIT): mean arterial pressure (MAP) ≤50 mmHg, systolic blood pressure (SBP) ≤50 mmHg, oxygen saturation (SPO_2_) ≤60% (17).

### Patient/graft survival

Recipient survival: calculated from the date of initial pancreas transplant to patient death (due to any cause). If death did not occur, the recipient was censored at their last known alive date.

Pancreas graft survival: calculated from the date of initial pancreas transplant to time of graft failure (as defined above).

Kidney graft survival: time of initial SPK transplant to time of permanent dialysis or recipient death.

### Technical aspects of organ procurement

#### DBD procurement

The DBD procurement was routinely performed in the same fashion. Warm dissection was performed as such that the pancreas was nearly entirely mobilized. Heparin was given 5 min prior to cannulation at a dose of 500 U/kg. Approximately 4–6 L of University of Wisconsin (UW) solution were flushed via the abdominal aorta, followed by cold dissection. The liver and pancreas were split *in situ* or procured en-bloc as per implanting surgeon preference. The allografts were packaged with the traditional cold static storage method.

#### DCD procurement

1,000 U/kg given prior to WLS. WLS occurred predominantly in the ICU or occasionally in the operating room (based on local hospital guidelines). After cardiorespiratory arrest, asystole was confirmed after a 5 min waiting period. In the operating room, the pancreas was procured with the standard rapid recovery technique. 4–6 L of UW solution were perfused via the abdominal aorta. The liver and pancreas were split *in situ* or en-bloc, depending on the implanting surgeon preference. We do not use a functional warm ischemia time. For the donor graft to be accepted, the time of WLS to time of perfusion had to be 30 min or less. Regional normothermic perfusion during procurement was not performed for any DCD procurement. DCD allografts were packaged with the static cold storage method.

### Transplant procedure

The pancreas allograft was prepared on the back table in the typical fashion. Donor iliac Y graft was anastomosed to donor pancreas splenic artery and superior mesenteric artery. Stapled ends of the duodenum were oversewn with 4-0 PDS suture. The kidney transplant was performed first and in the left iliac fossa, within the pre-peritoneal space or intraperitoneally depending on surgeon preference. The pancreas allograft was transplanted (after the kidney) intraperitoneally in the right iliac fossa with systemic venous drainage via the recipient vena cava. Donor iliac Y graft was most often anastomosed to the recipient right common iliac artery. For all cases, enteric drainage was performed by creating a Roux-en-Y limb of recipient small bowel that was anastomosed to the donor duodenum. Two-layer hand-sewn anastomosis was performed for all bowel anastomoses. We routinely placed a drain in the pelvis, near the tail of the pancreas. DCD transplants were performed in the same fashion as DBD transplants without any specific surgical modification for the DCD organs.

### Post-operative management

Immediately post-transplant, recipients were routinely transferred to the stepdown care unit as per protocol, whereas ICU admission occurred only if clinically indicated. Post-transplant immunosuppression predominantly consisted of thymoglobulin induction (5 mg/kg), maintenance with tacrolimus and mycophenolate mofetil along with a steroid induction, taper and maintenance. Post-operatively, recipients were initiated on standard deep vein thrombosis (DVT) prophylaxis and 81 mg of Acetylsalicylic acid (ASA) once recipient hemoglobin and hemodynamics were stable. Recipients of DBD and DCD grafts underwent the same post-management protocol.

### Statistical analysis

Patient demographics and clinical outcomes were described using descriptive statistics (mean or median including standard deviation). Chi-square test or Fisher's exact tests were used for binary and categorical variables. Kruskal–Wallis was used for continuous variables. Kaplan–Meier (KM) curves with log rank tests were used to identify unadjusted patient survival and graft survival times stratified by pancreas/kidney donor type (DCD vs. DBD). Numeric summaries of 8-year cumulative survival estimates were derived along with the respective 95% confidence intervals. The level of significance was set at *p* < 0.05 for all statistical analyses, and all reported *p* values reflect two-tailed tests. All data were gathered using Microsoft Excel spreadsheet software and GraphPad Prism 9 software was used to perform the statistical analysis.

## Results

Over the 10-year study period, 32 DCD donors were utilized for SPK (28 cases) and PAK (4 cases) transplantation. With a propensity matched cohort, 96 DBD cases consisting of 84 SPK and 12 PAK cases were compared to the DCD cohort. Baseline donor and recipient demographics are shown in Table 1, and peri-operative outcomes are shown in Table 2. DCD donors were significantly younger than DBD donors (22.7 vs. 28.7 years of age, *p* = 0.003), yet the BMI was similar between DCD and DBD donors (23.1 vs. 23.9, *p* = 0.35). No differences were found between DCD and DBD donor cause of death or gender.

**Table 1:** Donor and recipient demographics.

Demographics	DCD (*n* = 32)	DBD (*n* = 96)	*p*-value
Donor			
Age (mean ± SD), years	22.68 ± 6.71	28.65 ± 10.46	0.003
Cause of death (%)			
Anoxia	12 (37.5%)	47 (49%)	0.169
TBI	12 (37.5%)	25 (26%)	
CVA	3 (9.4%)	14 (16.1%)	
Cardiac	2 (6.2%)	1 (1%)	
Unknown	3 (9.4%)	9 (9.4%)	
Gender (%)			
Female	12 (37.5%)	30 (31.3%)	0.52
BMI (mean ± SD)	23.06 ± 3.67	23.93 ± 4.68	0.347
Recipient			
Age (mean ± SD), years	43.03 ± 8.72	44.60 ± 8.14	0.354
Gender (*n*) (%)			
Female	6 (18.8%)	37 (38.5%)	0.052
T1DM	25 (78.1%)	74 (77.9%)	1
T2DM	7 (21.9%)	21 (22.1%)	
BMI (mean ± SD)	25.89 ± 5.21	24.51 ± 3.96	0.117
Dialysis before transplant [*n* (%)]	30 (93.7%)	88 (91.6%)	0.523
Dialysis time (median ± SD), days	982 ± 1,097.5	1,062 ± 5,965.4	0.304
Days on the waiting list (median ± SD), days	334 ± 775	421 ± 438	0.354

TBI, traumatic brain injury; CVA, cerebral vascular accident; BMI, body mass index

**Table 2:** Recipient peri-operative outcomes.

	DCD (*n* = 32)	DBD (*n* = 96)	*p*-value
Procedure			
SPK	28 (87.5%)	84 (87.5%)	1
PAK	4 (12.5%)	12 (12.5%)	
Pancreas CIT (mean ± SD), min	579.64 ± 80.24	582.91 ± 115.63	0.902
Pancreas WIT (mean ± SD), min	33.79 ± 8.67	32.77 ± 9.3	0.641
CIT kidney (mean ± SD), min	433.83 ± 108.75	448.77 ± 101.8	0.553
WIT kidney (mean ± SD), min	34.13 ± 6.53	33.04 ± 8.79	0.588
Re-laparotomy	2 (6.3%)	10 (10.4%)	0.729
Vascular thrombosis	1 (3.1%)	7 (7.3%)	0.191
Arterial	0	3	
Venous	1	4	
Small bowel leak	3 (9.4%)	10 (10.4%)	0.866
Graft failure	10 (31.3%)	19 (19.8%)	0.223
Duodenal leak	1	4	
Acute rejection	3	1	
Chronic rejection	1	3	
Vascular thrombosis	0	2	
Acute pancreatitis	0	2	
DWFG	4	5	
Other	1	2	
Graft pancreatectomy	1 (3.1%)	9 (9.4%)	0.449
ICU length of stay (mean ± SD), days	0.91 ± 3.59	0.14 ± 0.94	0.056
Hospital length of stay (mean ± SD), days	13.50 ± 7.33	13.49 ± 6.93	0.994

SPK, simultaneous pancreas kidney; PAK, pancreas after kidney; CIT, cold ischemia time; WIT, warm ischemia time; DWFG, death with functioning graft.

Recipients of DCD and DBD allografts had no significant differences for age, gender, BMI, time on waitlist, time on dialysis, initiation of dialysis prior to transplant, transplant indications (T1DM vs. T2DM), and pre-transplant HBA1c levels. Male recipients were more common overall for both DCD and DBD organs. For both DCD and DBD recipients, T1DM was the main indication for transplant (78% of cases) while T2DM was indicated 22% of the time.

SPK transplant was performed in 87.5% of cases for both DCD and DBD groups. Recipients of DCD pancreas allografts had an overall vascular thrombosis rate of 3.1% (0 arterial, 3.1% venous) which was not significantly different to the DBD pancreas overall vascular thrombosis rate of 7.3% (3.1% arterial, 4.2% venous) (*p* = 0.19). Furthermore, there was no significant difference in CIT or WIT between DCD pancreas/kidneys and DBD pancreas/kidneys. There was no pancreas DGF or primary non-function in either the DCD or DBD groups. Small bowel leaks occurred at an incidence of 9.4% in DCD recipients vs. 10.4% in DBD recipients (*p* = 0.09). Graft pancreatectomy occurred at a rate of 3.1% (1/32) for DCD recipients vs. 9.3% in DBD recipients (9/96) (*p* = 0.44). In terms of infectious complications, there was no significant difference in outcome between rate of BK or cytomegalovirus (CMV) infection between groups (*p* = 0.83). The mean length of ICU stay for DCD and DBD recipients was 0.91 and 0.14 days, respectively (*p* = 0.06). Mean length of hospital stay was 13.5 days for DCD recipients and 13.49 days for DBD recipients (*p* = 0.99).

**Figure 1:**
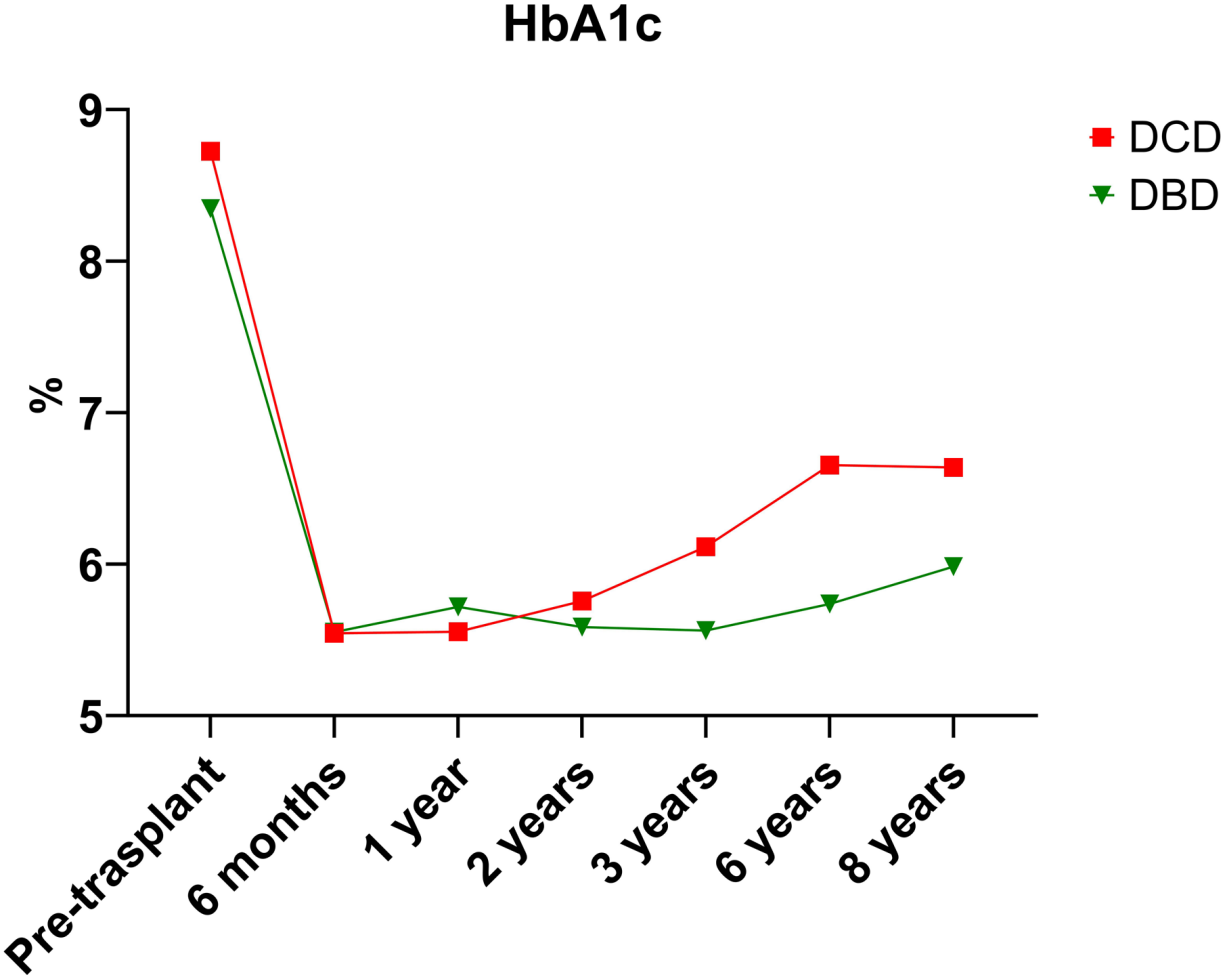
HbA1C.

Over the 8-year study period, 18.7% of DCD recipients experienced rejection compared to a DBD recipient rejection rate of 16.6% (*p* = 0.79). As expected, acute T-cell mediated rejection was the most common form of rejection encountered within both the DCD and DBD cohorts. The DCD pancreas graft failure rate over the 8-year study period was 31.3% compared to the DBD graft failure rate of 19.8% (*p* = 0.223). Acute rejection contributed to 3/10 cases of graft failure within the DCD group, compared to 1/19 cases leading to graft failure within the DBD group. Assessing 8-year post-transplant pancreas function, 18.8% of DCD recipients developed insulin resistance compared to only 6% of DBD recipients (*p* = 0.038). Those recipients who developed insulin resistance were started on oral hypoglycemics. If oral hypoglycemics were insufficient to manage glucose levels, insulin was initiated. DCD and DBD recipient HBA1c levels prior to transplant were similar at 8.73 ± 2.25 and 8.35 ± 1.81 (*p* = 0.426), respectively. At 6 months post-transplant DCD and DBD recipient HBA1c levels were 5.54 ± 0.69 and 5.55 ± 1.02 (*p* = 0.971). At 8 years post-transplant, DCD and DBD HBA1c levels were 6.64 ± 2.09 and 5.98 ± 1.75 (*p* = 0.393) (Table 3; Figure 1).

**Table 3:** Recipient post-transplant complications and outcomes.

	DCD (*n* = 32)	DBD (*n* = 96)	*p*-value
Infection			
BK	9 (28.1%)	31 (32.3%)	0.826
CMV	6 (66.7%)	15 (48.4%)	
CMV and BK	3 (33.3%)	10 (32.3%)	
Other	0	3 (7.5%)	
Kidney DGF	9 (28.1%)	6 (6.3%)	0.004
Glomerular filtration rate^a^			
1 month	60.47 ± 15.24	70.29 ± 20.35	0.016
3 months	70.14 ± 15.35	73.72 ± 18.83	0.364
6 months	65.31 ± 14.84	66.00 ± 19.32	0.861
1 year	61.72 ± 18.86	66.06 ± 19.36	0.296
2 years	61.71 ± 18.00	65.65 ± 22.34	0.403
3 years	64.27 ± 20.79	65.00 ± 23.16	0.888
6 years	67.60 ± 16.89	59.11 ± 23.53	0.202
8 years	60.82 ± 13.04	57.60 ± 21.21	0.645
Kidney failure	4 (12.5%)	8 (8.3%)	0.499
Pancreas rejection	6 (18.7%)	16 (16.6%)	
TCR	3	12	0.559
AMR	1	1	
Mixed	1	2	
Unknown	1	1	
Post-transplant T2DM	6 (18.8%)	5 (6.2%)	0.038
HBA1c			
Pre-transplant	8.73 ± 2.25	8.35 ± 1.81	0.426
6 months post-tx	5.54 ± 0.69	5.55 ± 1.02	0.971
1 year post-tx	5.55 ± 0.81	5.72 ± 1.19	0.498
2 years post-tx	5.76 ± 1.04	5.59 ± 0.71	0.333
3 years post-tx	6.12 ± 1.18	5.56 ± 0.70	0.006
6 years post-tx	6.65 ± 1.27	5.74 ± 0.77	0.002
8 years post-tx	6.64 ± 2.09	5.98 ± 1.75	0.393
Follow-up days (mean, SD)	2,151.9 ± 1,039.7	1,966.3 ± 1,036.8	0.382
Mortality (*n*) (%)	5 (15.6%)	8 (8.3%)	0.195

TCR, T-cell rejection; AMR, antibody mediated rejection; tx, transplant.

^a^ CKD-EPI creatinine equation 2021.

**Figure 2:**
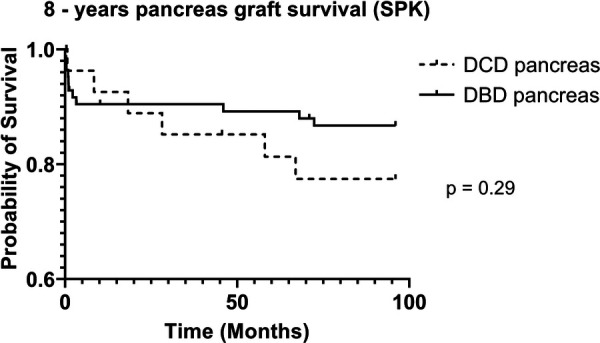
8-year recipient survival.

**Figure 3:**
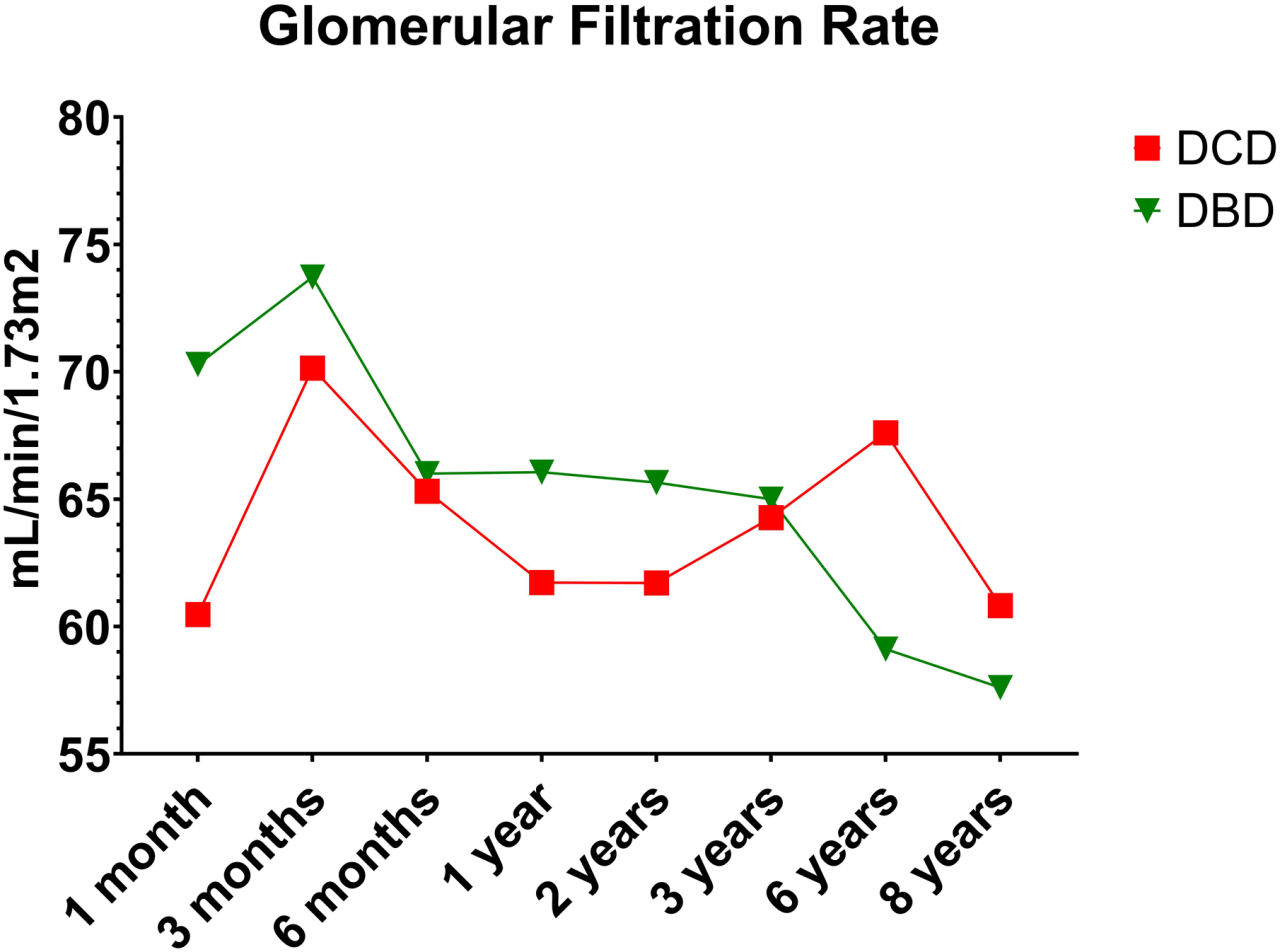
Glomerular filtration rate (using CKD-EPI creatinine equation 2021).

**Figure 4:**
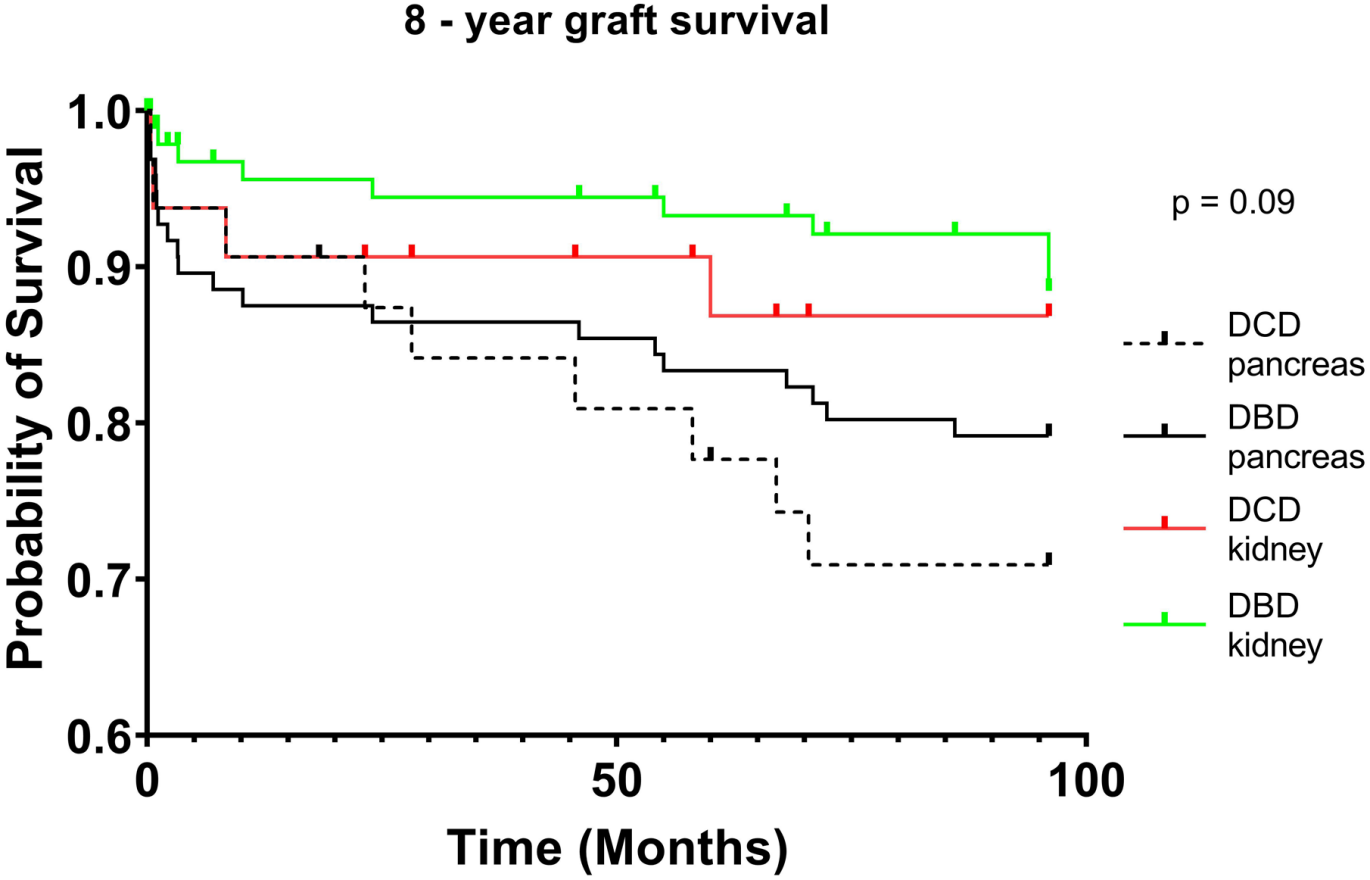
8-year graft survival.

**Figure 5:**
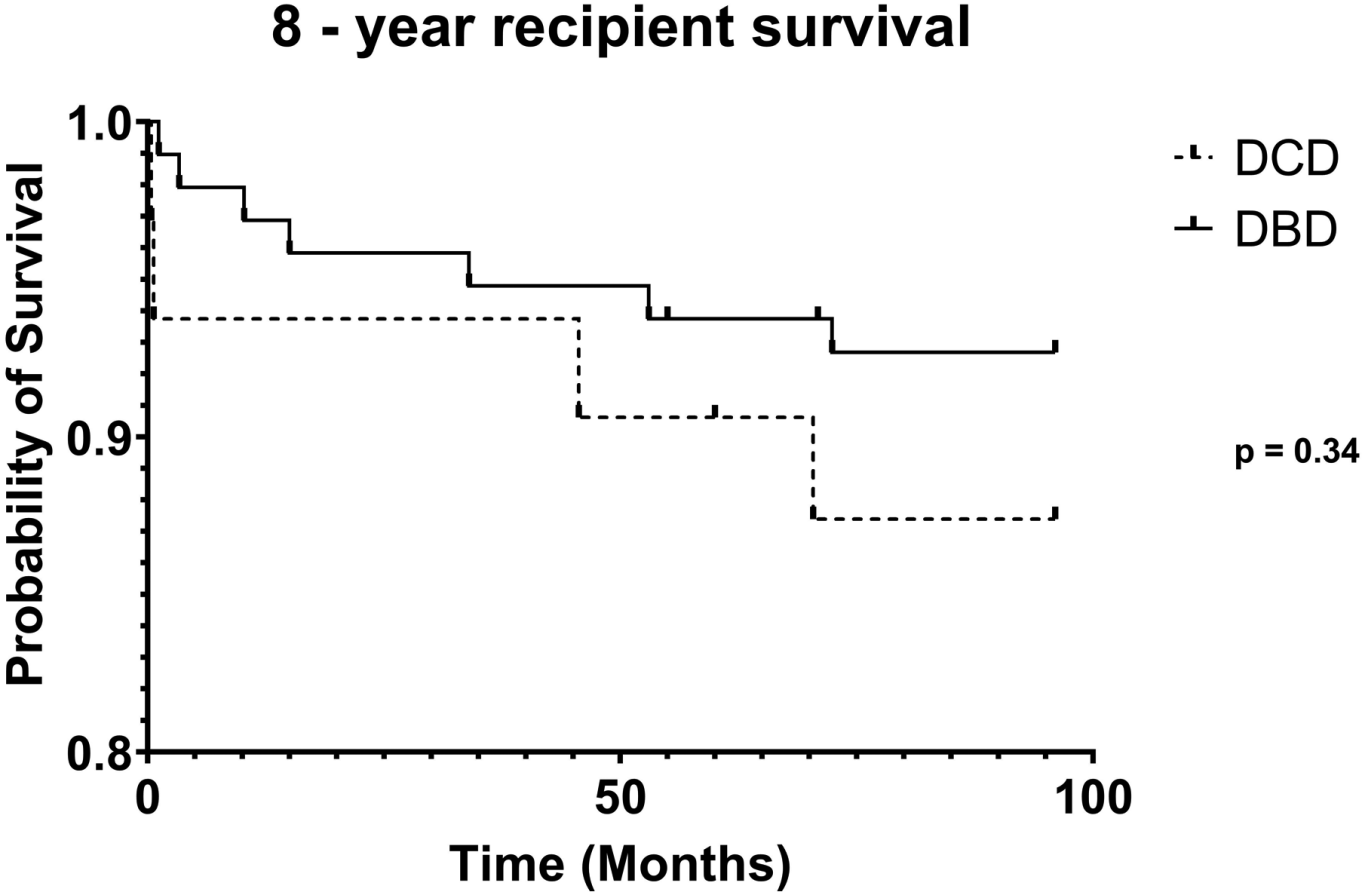
8-year pancreas survival (SPK).

**Figure 6:**
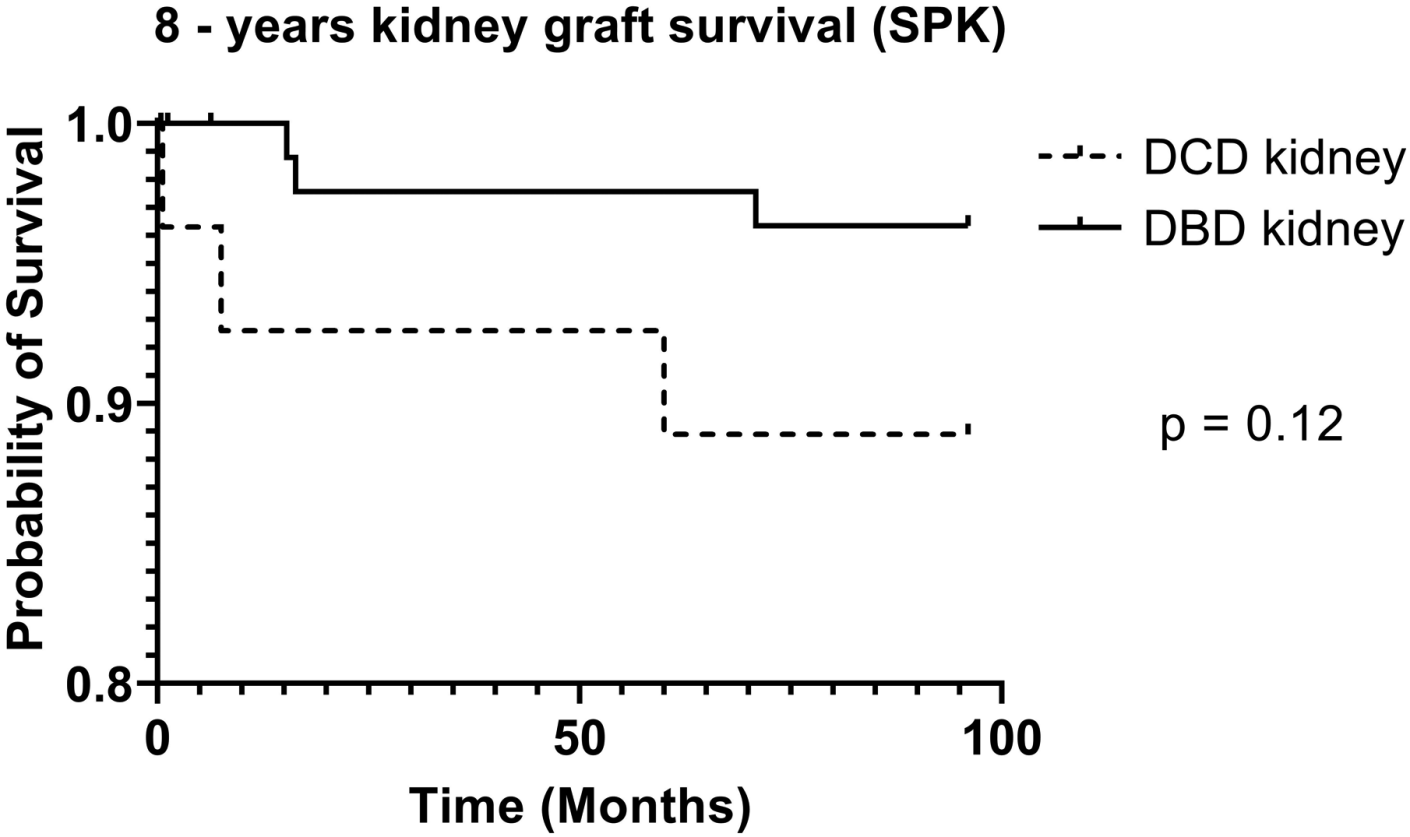
8-year kidney survival (SPK).

DCD recipients experienced a significantly higher rate of renal DGF (28.1% vs. 6.3%, *p* < 0.004). However, over the 8-year study period, the rate of kidney failure was similar (12.5% vs. 8.3%, *p* = 0.5). Overall, there was no significant difference in 8-year recipient survival and glomerular filtration rate for those receiving DCD or DBD grafts (87.4% vs. 92.7%, *p* = 0.348) (Figures 2, 3). When comparing overall graft outcomes by donation status, there was no significant difference in graft survival (*p* = 0.09) (Figure 4). Comparing SPK transplants, there was no difference in 8-year pancreas graft survival between DCD and DBD grafts (77.4% vs. 86.7%, *p* = 0.292) (Figure 5), nor was there any difference in 8-year kidney graft survival between DCD and DBD grafts (88.9% vs. 96.3%, *p* = 0.129) (Figure 6).

## Discussion

This single center, Canadian retrospective study demonstrates no significant differences in 8-year recipient survival between SPK DCD and DBD groups, along with no significant differences in graft survival between DCD and DBD pancreas and kidney grafts. Herein, we provide new data reconfirming previously published reports that DCD SPK transplantation has similar graft and patient survival compared to DBD SPK transplantation (18–21). Despite the retrospective nature of this study, there are several strengths of the paper that differentiate it from other previously published reports. First, this is a single center, propensity matched study, matched for donation status (DCD vs. DBD), transplant type (SPK vs. PAK) transplant date, and recipient indication. Second, DCD and DBD SPK transplant recipients within the study underwent identical management pathways (operative technique, post-op destination, immunosuppression, general post-operative care) limiting heterogeneity between groups. Third, we had 100% patient follow-up, report on 8-year follow-up data and collected yearly post-operative HBA1c levels.

In 2005, Fernandez et al. (18) published the first report of long-term outcomes of SPK transplantation comparing 37 DCD to 576 DBD transplants over a 10-year period. This was a non-propensity matched, retrospective study, where the authors reported similar 5-year recipient survival rates between DCD and DBD groups (91.5 vs. 89.1%, *p* = 0.85). Five-year pancreas DCD graft survival was also in line with pancreas DBD graft survival (72.2 vs. 78.9%, *p* = 0.18), as was 5-year DCD kidney graft survival compared to DBD graft survival (84.7% vs. 81.6%, *p* = 0.65). In 2014, Siskind et al. (22) reported data on adult pancreas transplant outcomes from the UNOS database, comparing 320 DCD vs. 20,448 DBD donors. The authors found no significant differences in respective DCD and DBD 10-year post-transplant graft survival (55.2% vs. 48.7%, *p* = 0.280) or recipient survival (80.9 vs. 72.1%, *p* = 0.061). Similarly, no differences were identified at 15-years post-transplant for graft survival (22.9% vs. 33.7%, *p* = 0.362) and recipient survival (29.7% vs. 59%, *p* = 0.057). The strengths of this study are the large numbers of patients, and second, the extended follow-up of 15-years post-transplant. However, the study is limited by its retrospective nature and the biases associated with large databases. In 2018, from the Netherlands, Kopp et al. (23) compared 21 DCD to 83 DBD pancreas transplants between 2011 and 2015 (with a short 2-year follow-up) and found no significant differences between DCD and DBD graft survival or recipient survival. Similar to the UK, a functional donor WIT was used, and no antemortem interventions were performed.

In 2021, a recent UK registry analysis demonstrated on both univariate and multi-variate analysis no statistical difference in 5-year graft or patient survival between DCD and DBD donor groups (13). The authors identified that DCD donors were significantly younger (30 vs. 37, *p* < 0.01), had a lower rate of stroke (as a cause of death), had lower terminal creatinine and had lower pancreas risk index scores compared to DBD donors. However, DCD recipients had significantly shorter waitlist time to transplant, pancreas CIT, and a higher rate of induction with a T-cell depleting agent compared to DBD recipients.

Our study also demonstrated that DCD donors were significantly younger compared to DBD donors (22.7 vs. 28.7 years of age, *p* = 0.003). Both the DCD and DBD ages were much younger in our study compared to the UK analysis. This likely reflects the more aggressive donor selection in the UK. In addition, in our study, no differences were found between donor cause of death, donor BMI, recipient time on dialysis, or days on the waiting list, nor any differences were found in pancreas CIT (DCD 9.65 h vs. DBD 9.7 h, *p* = 0.90) and anastomotic time (Table 2).

Our reported pancreas CIT is also shorter compared to the pancreas CIT reported by Muthusamy et al. from their UK registry analysis (12.30 h for DCD vs. 12.32 h for DBD grafts) (20). Interestingly, the DCD and DBD age (28 and 37, *p* < 0.01) reported from this analysis was younger compared to two other reports (13, 24). Shahrestani et al. (24) found a smaller age gap between donor groups with a mean DCD age of 26 and DBD age of 27, while Callaghan et al. (13) found a much higher DCD age of 30 and DBD age of 37.

Fernandez et al. (18) demonstrated that DCD SPK transplant recipients have a significantly higher rate of renal DGF compared to DBD SPK recipients (24.3% vs. 5.2% *p* = 0.0002). Callaghan et al. similarly identified higher rates of DGF from DCD donors (25% vs. 11%, *p* < 0.001) without any increased risk of renal failure compared to DBD donor grafts (13). This is in keeping with the rate of DGF of 28.1% from DCD kidney grafts vs. a DGF rate of 6.3% from DBD donors observed within this study. The higher incidence of renal DGF within our DCD cohort did not result in a significantly increased rate of kidney failure as compared to the DBD cohort (12.5% vs. 8.3%, *p* = 0.49).

The 8-year post-transplant pancreatic vascular thrombosis rate amongst our DCD cohort was low at 3.1% while the DBD pancreatic vascular thrombosis rate was 7.3% (*p* = 0.19). There were no cases where vascular thrombosis led to graft failure within the DCD group. The UK registry analysis by Callaghan et al. (13) also did not identify any difference in early pancreas graft loss due to vascular thrombosis between donor types. The low DCD pancreatic vascular thrombosis rate witnessed in our study supports the findings of a previously published, smaller, non-matched retrospective Canadian study which demonstrated that the rate of vascular thrombosis is not increased in DCD grafts compared to DBD grafts (15). The thrombosis rate exhibited within our study is also in keeping with the overall thrombosis rate of 6% reported by Bellingham et al. (21). Contrarily, Muthusamy et al. (20) have reported an increased numeric 1-year vascular thrombosis rate in DCD pancreata of 8% compared to 5% within DBD pancreata, however this was non-significant. In addition, a meta-analysis performed by Shahrestani et al. (24) found an increased DCD pancreas vascular thrombosis rate compared to the DBD cohort with an odds ratio (OR) of 1.67 (*p* = 0.006). Subgroup analysis identified that the use of antemortem heparin for DBD and DCD donors had no significant difference in vascular thrombosis rates (OR 1.29, *p* = 0.62). It has also been demonstrated that a higher DCD thrombosis rate does not necessarily lead to lower graft or recipient survival (14).

At our institution, both DCD and DBD donors receive premortem heparin which may be a contributing factor to the low and non-significant differences of vascular thrombosis between donor groups. In addition, the young DCD donor age and 30-min DCD WIT limit may have potentially influenced the low vascular thrombosis rate seen within our study.

The graft failure rate within this study was similar between the DCD and DBD groups at 31.1% and 19.8% (*p* = 0.22). Of note, the most common cause of graft failure within both DCD and DBD donor groups was death with a functioning graft. The second most common cause of graft failure in the DCD group was acute cellular mediated rejection, followed by duodenal leak. Within the DBD group, the second most common cause of graft failure was the occurrence of a duodenal leak followed by chronic rejection. Vascular thrombosis led to DBD graft failure in only 2/19 cases. The 8-year pancreas rejection rate was 18.7% in the DCD cohort compared to 16.6% in the DBD cohort, both of which were lower than the 10-year pancreas rejection rates reported by Bellingham et al. (21) (27% DCD vs. 20% DBD rejection rate).

Previous studies from North America are limited by small study numbers and/or are no longer recent. Although the UK pancreas DCD experience is quite extensive, there exist several differences in practice between the UK, USA and Canada, which may limit generalizability of outcomes. In the UK, donor pancreas offers are at a national level, whereas in Canada offers are primarily at a provincial level, followed by a national level. In Canada, there is essentially no national competition for pancreas donors, as the large geographic distances preclude routine sharing of pancreas organs across provinces. Contrary to the UK, at our institution we decline DCD pancreas donors with a total WIT beyond 30 min, do not use a functional WIT, and premortem heparin is given for all DCDs (as per institutional protocol). Within Ontario, only two centers (including ours) perform pancreas transplantation. With a large general population of approximately 14 million people and limited center competition, our ability to highly select both DCD and DBD donors for pancreas transplantation is of unique benefit. This may help explain the young donor population within both DCD and DBD groups. In addition, procurement of pancreas grafts is without exception routinely performed by our own institution's surgeons thereby limiting variability of technique, and potential for donor procurement complications.

Limitations of this study are in keeping with the inherent limitations associated with retrospective analysis. For example, inaccurate data entry or confounders cannot be accounted for within the retrospective design.

## Conclusion

This propensity matched, single center, retrospective Canadian study demonstrates that within pancreas transplantation, use of DCD allografts results in similar 8-year recipient and graft survival in comparison to DBD allografts. There is an increased incidence of renal DGF for DCD recipients yet there is no difference in renal failure outcomes between recipients of DCD and DBD donors. The vascular thrombosis rate was similarly low between DCD/DBD grafts without any significant differences. At 8-years post-transplant, DCD recipients had a higher rate of developing T2DM compared to DBD donors; however, there is no significant difference in HBA1c levels between DCD and DBD recipients. The use of DCD allografts is safe for pancreas transplantation and can provide comparable outcomes to DBD pancreas transplantation. Increased utilization of well selected DCD grafts for pancreas transplantation is an appropriate alternative to help expand the donor pool and reduce recipient waitlist times, without compromise to recipient longevity and graft survival.

## Data Availability

The original contributions presented in the study are included in the article/Supplementary Material, further inquiries can be directed to the corresponding authors.
